# Continuous Critical Respiratory Parameter Measurements Using a Single Low-Cost Relative Humidity Sensor: Evaluation Study

**DOI:** 10.2196/47146

**Published:** 2023-10-25

**Authors:** Fabrice Vaussenat, Abhiroop Bhattacharya, Julie Payette, Jaime A Benavides-Guerrero, Alexandre Perrotton, Luis Felipe Gerlein, Sylvain G Cloutier

**Affiliations:** 1 Department of Electrical Engineering École de Technologie Supérieure Montreal, QC Canada

**Keywords:** relative humidity sensor, design, develop, development, tidal volume, pulmonary volume, COPD, pulmonary, respiratory, sensor, sensors, wearables, humidity, medical device, development, breathing, wearable, ventilation, air

## Abstract

**Background:**

Accurate and portable respiratory parameter measurements are critical for properly managing chronic obstructive pulmonary diseases (COPDs) such as asthma or sleep apnea, as well as controlling ventilation for patients in intensive care units, during surgical procedures, or when using a positive airway pressure device for sleep apnea.

**Objective:**

The purpose of this research is to develop a new nonprescription portable measurement device that utilizes relative humidity sensors (RHS) to accurately measure key respiratory parameters at a cost that is approximately 10 times less than the industry standard.

**Methods:**

We present the development, implementation, and assessment of a wearable respiratory measurement device using the commercial Bosch BME280 RHS. In the initial stage, the RHS was connected to the pneumotach (PNT) gold standard device via its external connector to gather breathing metrics. Data collection was facilitated using the Arduino platform with a Bluetooth Low Energy connection, and all measurements were taken in real time without any additional data processing. The device’s efficacy was tested with 7 participants (5 men and 2 women), all in good health. In the subsequent phase, we specifically focused on comparing breathing cycle and respiratory rate measurements and determining the tidal volume by calculating the region between inhalation and exhalation peaks. Each participant's data were recorded over a span of 15 minutes. After the experiment, detailed statistical analysis was conducted using ANOVA and Bland-Altman to examine the accuracy and efficiency of our wearable device compared with the traditional methods.

**Results:**

The perfused air measured with the respiratory monitor enables clinicians to evaluate the absolute value of the tidal volume during ventilation of a patient. In contrast, directly connecting our RHS device to the surgical mask facilitates continuous lung volume monitoring. The results of the 1-way ANOVA showed high *P* values of .68 for respiratory volume and .89 for respiratory rate, which indicate that the group averages with the PNT standard are equivalent to those with our RHS platform, within the error margins of a typical instrument. Furthermore, analysis utilizing the Bland-Altman statistical method revealed a small bias of 0.03 with limits of agreement (LoAs) of –0.25 and 0.33. The RR bias was 0.018, and the LoAs were –1.89 and 1.89.

**Conclusions:**

Based on the encouraging results, we conclude that our proposed design can be a viable, low-cost wearable medical device for pulmonary parametric measurement to prevent and predict the progression of pulmonary diseases. We believe that this will encourage the research community to investigate the application of RHS for monitoring the pulmonary health of individuals.

## Introduction

### Theory

In 2020, respiratory disorders impacted approximately 550 million individuals globally and caused 4 million annual deaths. The COVID-19 pandemic led to an increase in health care expenditure, particularly in the field of respiratory diseases [1]. The pandemic expedited the development of respiratory diseases, and despite persistent respiratory and neurological problems, many patients have been discharged [2,3]. Simultaneously, obstructive sleep apnea (OSA) [4] affects a significant proportion of adults and is related to increased morbidity and mortality worldwide.

These significant global health care issues warrant the pursuit of solutions to prevent and optimize health care pathways [5]. Lung volume measurement is especially important for patients with respiratory diseases [6,7], with OSA and under ventilation [8], or in intensive care units (ICUs) [9]. Spirometers can evaluate breathing in patients with asthma or chronic obstructive pulmonary disease (COPD) [10]. Patients with OSA using an automatic positive airway pressure (A-PAP) or continuous positive airway pressure (C-PAP) device must be awake to execute deep breathing (inhale and exhale) to be tested with a spirometer [11]. Wearable devices that assess respiratory rate (RR) using validated methods have been developed recently [12-15], but they do not measure lung volume [16]. Thus, a wearable device that gives comprehensive lung volume data to improve quality of life, monitor remotely, and avoid respiratory disease progression would be highly desirable [17,18].

In this work, we present a simple and inexpensive sensor platform that can be used to quantify pulmonary inspiration, expiration, and lung volumes. Our device uses a relative humidity sensor (RHS) to detect breathing and calculate tidal volumes (TVs), expiratory reserve volumes (ERVs), and inspiratory reserve volumes (IRVs) [19]. To the best of our knowledge, the innovative aspects of this study are the direct measurement of respiratory cycles and the exact derivation of TD, ERV, IRV, and vital capacity (VC) data from the calculation of the breathing surface [16,20].

### Prior Work

There are numerous techniques for assessing respiratory function and detecting lung disorders such as COPD and asthma [1]. Pulmonary function tests need accurate breathing volume and flow measurements using a basic spirometer [21], requiring the patient to inhale deeply and then expel as forcefully as possible via the mouthpiece over a period of time. This is an inexpensive, noninvasive test that can be administered in a medical facility or at home. However, the requirements of this test can still be hard for some patients who cannot fully empty their lungs during the procedure. This limits the usefulness of this well-known diagnostic tool [22]. Additionally, laboratory blood tests can be used to evaluate respiratory health. However, because this is an intrusive procedure, it cannot be used to indefinitely monitor patients outside of hospitals.

The number of rib cage movements per minute is another crucial indicator that indicates respiratory and heart health, via RR [23,24]. RR monitoring can be accomplished using ICU-specific equipment [25]. Mathematical correlations of photoplethysmography (PPG) and electrocardiography (ECG) data yield accurate RR values [26-28]. Numerical methods estimate the RR from PPG and ECG using the following 3 physiological modulations of breathing: amplitude modulation, frequency modulation, and baseline wander [29,30]. The noise in the PPG and ECG signals affects the accuracy of these RR measures [31], and considerable signal processing is needed to extract meaningful information from the noise and improve the measurements [32,33].

Measuring RR alone cannot assess lung capacity, a critical indication of COPD status and development [34]. Respiratory depression can be detected by lung volume measurements such as TV, ERV, IRV, and VC. Pulmonary function tests measure lung volume, capacity, flow rates, and gas exchange. Spirometry has low accuracy and significant latency and cannot be utilized during sleep [35]. Plethysmography, which measures intrathoracic gas during airflow obstruction, is used to calculate lung volume [36]. Modern wearable devices like CO_2_ gas sensors analyze CO_2_ or O_2_ fluctuations during inspiration and expiration to estimate lung capacities [37]. Computed tomography radiography, which is invasive and time-consuming, is another option [38,39].

**Figure 1:**
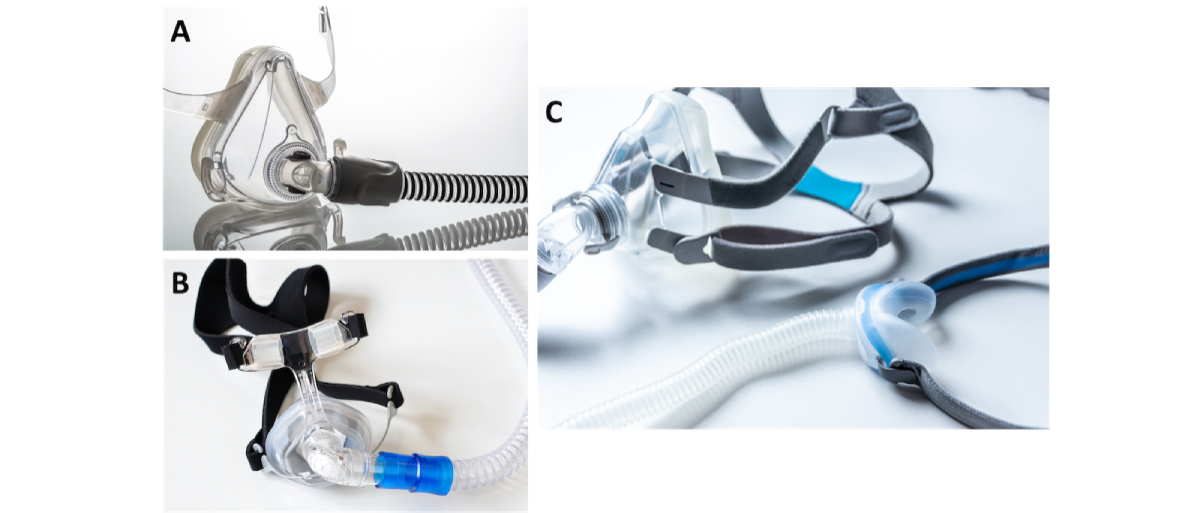
Commercial face masks typically used (A) in intensive care units with the (B) ventilation connector including respiratory rate, humidity, and pressure measurement sensors and (C) at home by patients with obstructive sleep apnea. All products are manufactured by ResMed Ltd.

A much easier method measures the RR directly from the moisture content of the breath [20,40,41]. Patients are typically attached to various monitoring devices in ICUs and during surgery to continually monitor their pulse, blood pressure, breathing rate, and oxygen saturation. Typically, humidity sensors are included in the tube adaptor entry of respirator face masks (Figure 1) [42].

Continuously measuring the humidity of the exhaled air provides an accurate measurement of the patient's RR. In addition, it was recently shown that TV might potentially be effectively estimated from surface measurement during the normal breathing cycle [43]. Exhaled air has a relative humidity (RH) of 100% and is saturated with water [44]. This exhaled humidity is a function of pulmonary capacity and is proportional to RR and lung volume [20,45]. Most crucially, atmospheric pressure, external temperature, and sex-dependent fluctuations alter the signal amplitude's maximum RH content [46].

A huge network of internet-connected objects, including Bluetooth Low Energy devices, sensors, and global positioning systems, is the goal of emerging Internet of Things (IoT) paradigms [40,41]. Medical IoT devices that use cloud compute power could improve chronic respiratory illness detection and therapy. In medical IoT devices, embedded electronics like accelerometers [17,42-44] and temperature sensors [45,46] are used in new ways. Using a chest-mounted belt, an accelerometer may record rib cage movement to determine breathing rate. The inhalation-exhalation temperature differential can also be monitored [47]. We can estimate the key pulmonary indicators from studying the relationship among temperature, pressure, and humidity of a person [48]. The only IoT-compatible methods for measuring lung capacity are somewhat sophisticated spirometry [49], capnometry [50], and impedance pneumography devices [51,52]. In this new paradigm, data collection occurs simultaneously in real time and over an extended period of time, enabling a move from a reactive treatment strategy to an early warning and detection mode that maximizes results while minimizing the related human and financial costs [53].

### Hypothesis

**Figure 2:**
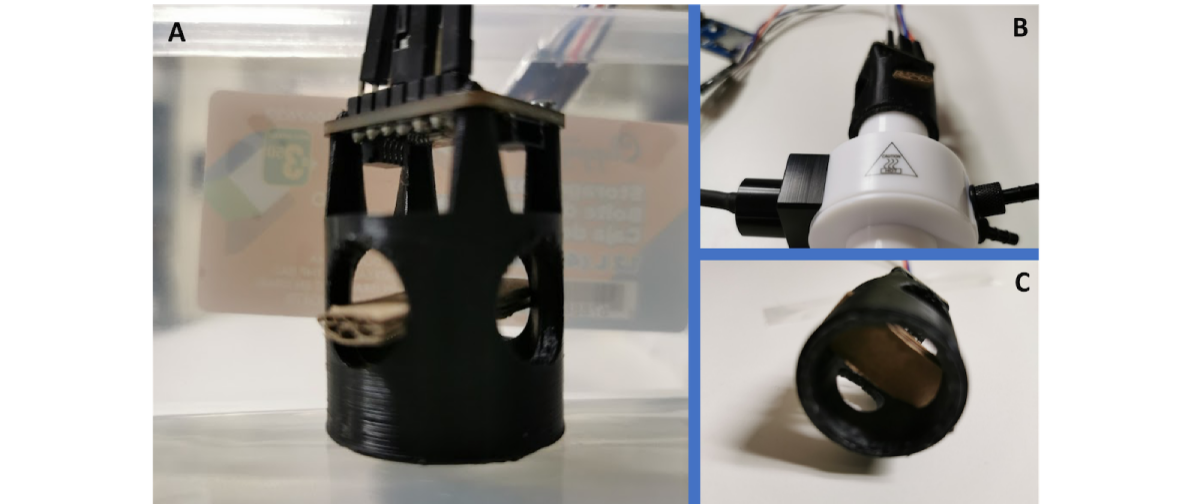
Humidity sensor adapter connected to the obstructive sleep apnea face mask to reduce the amount of trapped moisture: (A) outside view of the airflow adapter with the reduction and humidity sensor, (B) inside view of the airflow adapter with the reduction and humidity sensor, and (C) airflow adapter with the reduction and humidity sensor and the heater connected.

The key hypothesis of our work was that the changes in RH during breathing provide an indication of pulmonary disorders in a patient. Moreover, we also believed that the change in RH during deep inhale-exhale cycles of breathing can be used to measure pulmonary volume, especially TV, ERV, and IRV. The combination of measurements provides the total pulmonary VC. To prove our hypothesis, we designed a low-cost wearable device that uses a single RHS to provide accurate and sophisticated lung volume (TV, ERV, IRV, and VC) and RR measurements. To validate our strategy, we incorporated the electronic prototype into an OSA face mask with our own flow adapter (Figure 2), which is designed to prevent moisture retention and keep the sensor close to the mouth and nose.

The gap between the mask and humidity sensor was defined to avoid saturation of the humidity sensor. In fact, we tested different tube sizes to support the sensor, so as to avoid phenomena saturation during deep exhalations. We based our adapter on the venturi effect [54]. Various flow measurements were carried out to create this adapter, from which the sensor is attached and onto which the mask is fitted. This method is also used to easily change the mask from one person to another.

We used statistical analysis methods including Bland Altman, 1-way ANOVA, and box plots to validate our results. The preliminary results of the experiments with multiple participants showed that our hypothesis was correct, and the same was corroborated by the statistical tests. With more participants and sophisticated models, we will be able to classify pulmonary disorders based on changes in RH.

## Methods

### Experimental Setup

**Figure 3:**
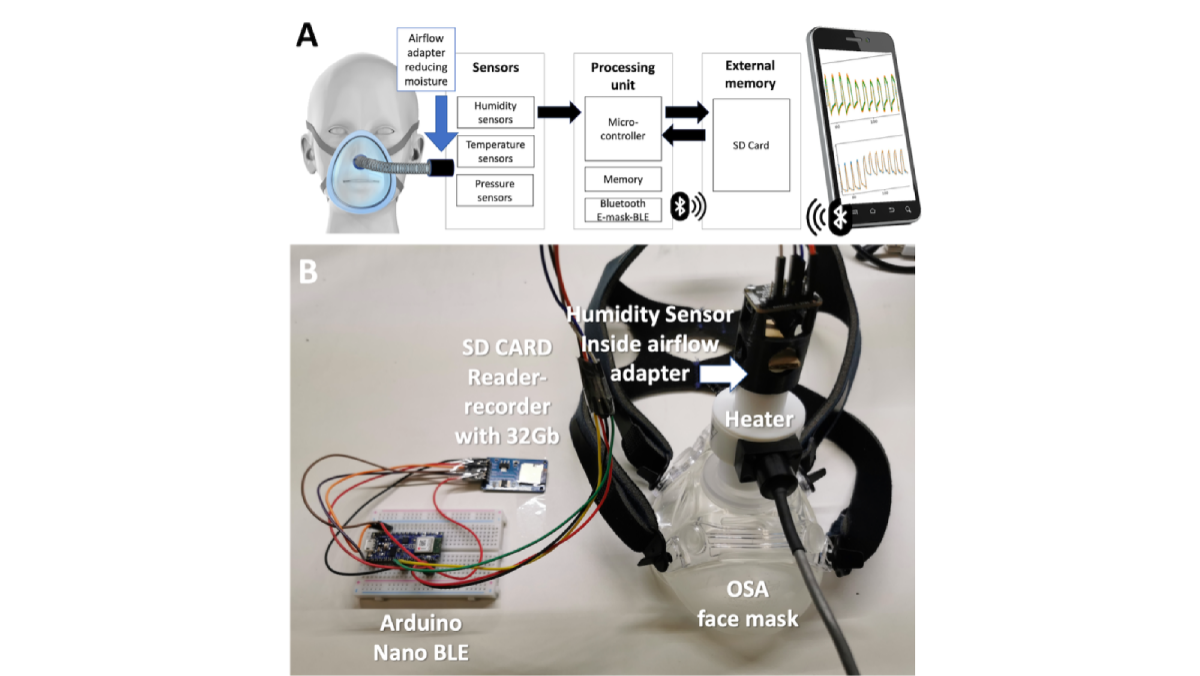
(A) Schematics of the proposed health care Internet of Things architecture and (B) our prototype. BLE: Bluetooth Low Energy; OSA: obstructive sleep apnea.

We used the humidity sensor to estimate lung volumes, a key respiratory function, by monitoring inspiratory and expiratory humidity [47]. Figure 3 shows the electronic prototype inserted into an OSA face mask to keep the sensor close to the mouth and nose. The BME280 humidity sensor was protected from face mask moisture by a 3D-printed airflow adapter. Bluetooth data transmission and SD card data recording were enabled by an Arduino Nano Bluetooth Low Energy interfaced to the sensor. Figure 3A provides a schematic overview, and Figure 3B depicts our prototype. We developed the device using a proven health care IoT architecture strategy that combines data acquisition, low energy, and embedded systems [55]. Figure 3A shows the medical-compatible BME280 sensor measuring RH, ambient temperature, and barometric pressure [56,57]. Additionally, it has quick start-up and recovery times, with a 63% recovery occurring in roughly 1 second [58]. BME280 is a widely used, commercial humidity sensor that retains a long-term stability of ±0.5% RH per year and a low signal-to-noise ratio of 0.02% RH, as provided in the sensor's data sheet [58]. The range of the RH measurement at an overall level is 89 (SD 7.8).

The sensor's embedded data preprocessing is enabled by an NRF52832 microcontroller unit used in other medical devices [59]. The microcontroller unit stores data on the SD card for later retrieval when a Bluetooth connection is unavailable. The smartphone's wireless connection and real-time data recording app were developed in Android Studio. The IEEE 754 standard requires converting the data transmitted via Bluetooth from HEX to float [60]. Data cleaning, processing, and automated parameter calculation were done via Python scripting.

We worked on the raw data to highlight all possible anomalies and artifacts. As the humidity sensor is placed on the front of the mask, separated by a support to prevent saturation of the sensor during deep exhalation, no movement artifacts were detected. The only artefacts measured were those associated with breathing, which can sometimes present saccades that highlight the absence of ventilatory recovery.

**Figure 4:**
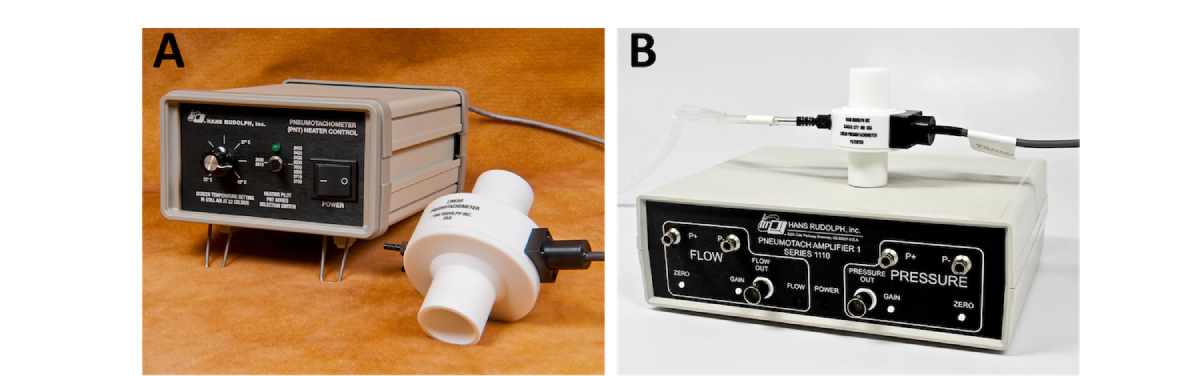
Commercial pneumotach (PNT) used for reference baseline measurements: (A) PNT controller and heater and (B) PA-1 PNT amplifier. The photos were taken from Hans Rudolph website [61].

A standard commercial breathing monitoring device (pneumotach [PNT]; Hans Rudolph) was used to independently calculate the lungs' volume and RR for comparison with our sensing platform's results. Figure 4A shows the PNT controller and heater [61]. Figure 4B shows the Hans Rudolph PA-1 PNT Amplifier [62]. Standard PNT devices monitor respiratory parameters at 1 kHz [62,63].

**Figure 5:**
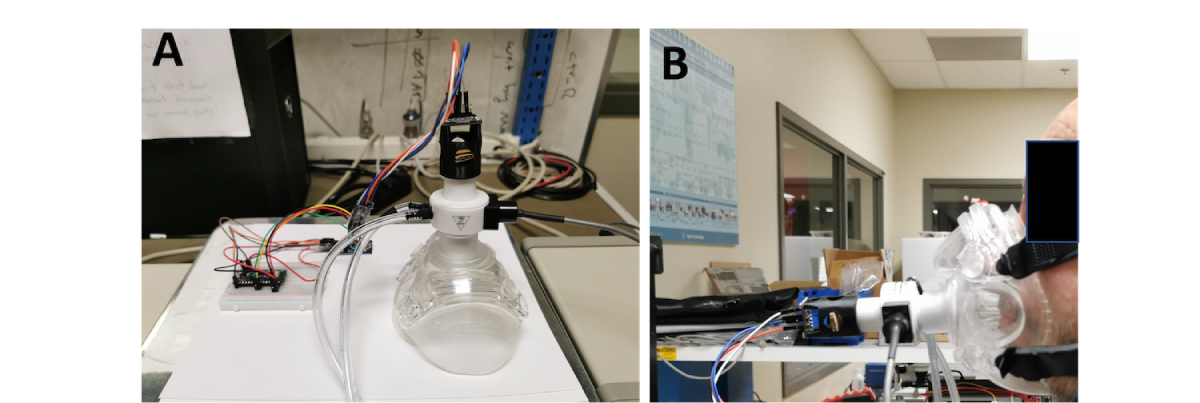
Testing procedures, including the (A) global measurement setup and (B) face mask connected to the heater and relative humidity sensor adapter.

For best results, our sensor should be placed behind the PNT heater using an adapter to lower face mask humidity. This adaptor in the face mask allowed precise humidity measurement during respiration, enhancing sensor sensitivity and limiting saturation. This design integrates our humidity measuring device into the surgical face mask and connects via the PNT, as shown in Figures 4 and 5. All participants were instructed to breathe deeply every 30 seconds to determine TV, IRV, and ERV, then they were instructed to breathe normally for the rest of the 15-minute test. The VC is the sum of the IRV and ERV parameters. The RH sensor sampled data at 5 kHz, and the PNT sampled data at 1 kHz during the tests.

### Recruitment

All study participants, comprising 5 men and 2 women, were healthy and had no preexisting respiratory conditions. Their mean height was 171 (SD 10) cm, and their mean weight was 75 (SD 20) kg. The mean BMI was 25 (SD 4) kg/m^2^. The mean age of this population was 38 (SD 8) years. All participants were tested under identical conditions under medical supervision.

The experiment was conducted according to a specific protocol that ensured “identical” conditions for all participants involved. First, implementation of the mask was dealt with in 2 parts. Each participant was given a dedicated, single-use mask. The sensor part of the mask, affixed to the mount, was reusable and did not need to be cleaned. To monitor its operation, a data acquisition system was used to confirm that the sensor maintained the same humidity saturation as the room environment.

All measurements were taken at the same RH, in the same location, and under the same automated climatization conditions. These conditions included temperature, humidity, and atmospheric pressure. To ensure this uniformity, all measurements were conducted on the same day.

Regarding the selection of participants, they were chosen based on specific criteria to ensure their homogeneity. Participants had no respiratory history and were in good physical condition. They all used the same seated position and the same chair and were exposed to the same environmental conditions as other participants.

In terms of equipment setup, the equipment was placed uniformly for all participants. A preliminary step in the protocol confirmed that the baseline conditions (temperature and humidity in the room) were the same for all participants before starting the experiment. A new mask was used for each measurement to eliminate any risk of cross-contamination or interference.

Physical parameters such as height, weight, and blood pressure were measured before data collection, and all participants were allowed 5 minutes of relaxation before starting.

**Ethics Approval**

The experiments presented in this paper were approved by the research ethics committee at École de Technologie Supérieure (approval number: H20230603).

### Statistical Analysis

We compared the means and IQRs from the commercial PNT and our prototype using a box plot. We also compared the deep breathing area (DBA) and RR series using a 1-way ANOVA [64]. The hypothesis was tested with an optimal *P* value of .05 in the ANOVA analysis. DBA and RR Bland-Altman charts were plotted against the PNT for all participants.

### Methodology

A cleaning protocol was used for the mask before each measurement to remove contamination risks. The following method derives respiratory parameters from sensor-measured RH and PNT: According to the experimental section, the RH was measured throughout the 15-minute test. Counting maxima over a time segment gives the RR. We calculated TV by calculating the area under the curve between 2 consecutive minima using normal breathing data. All lung volumetric parameters were calculated using the rectangle method, which is presented in mathematical form in equation 1 [65].



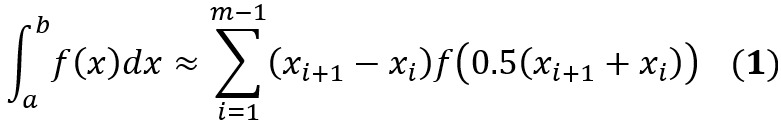



where *i* indicates the sample number, *x* indicates the time duration of measurement in seconds, *f* indicates the function to compute the RH value at time *x*, and *m* indicates the total number of samples.

Deep inhalation and exhalation areas determine IRV and ERV parameters, and the VC is the sum of both. The beginning and end of each deep inspiration and expiration cycle were indicated by a sign change in the signal's second derivative [5]. Counting minima in beats per minute over a chosen time can also determine the RR, as shown in equation 2.



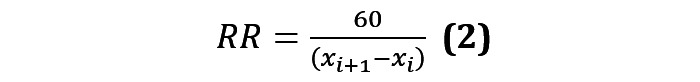



where *x* indicates the time duration of thr measurement in seconds.

**Figure 6:**
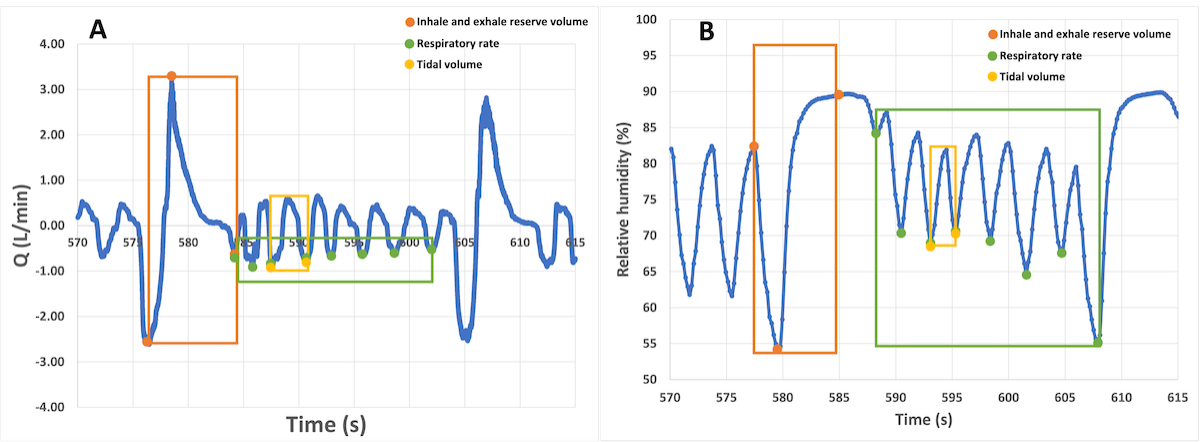
Respiratory parameters calculations from the (A) commercial pneumotach (PNT) recordings and (B) relative humidity sensor–based prototype.

Indeed, RR and TV have been used to validate respiratory parameters. As shown in Figure 6, lung volumes and RR were calculated by measuring deep inhalation and exhalation breathing and the respiratory cycle over 1 minute. The room temperature remained at 21.5 °C. External humidity control in the test room was turned off because the mask uses a heater to control humidity.

Our prototype's reset button synchronized data collection timers with the PNT. A computer saved the PNT's benchmark respiratory parameters. Volume capacity is indicated by the areas of the orange rectangle in Figure 6A, which represents the closed DBA signal that begins with inhalation and ends with deep exhalation. RH and PNT had the best correlation (R=0.84) when calculating the area using the rectangle method. The triangle method had a lower correlation coefficient (R=0.40).

## Results

### Bland-Altman Analysis

**Figure 7:**
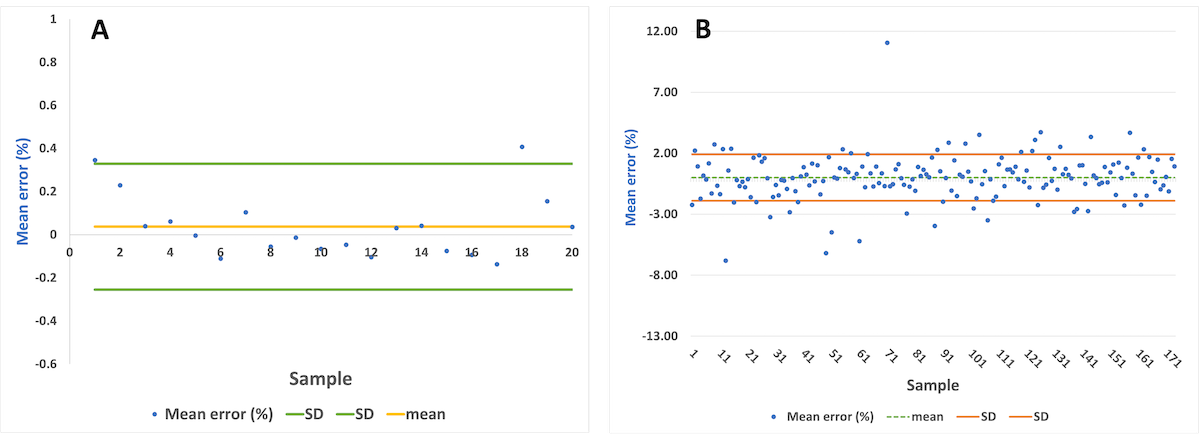
The Bland-Altman plot and calculations comparing the values from the relative humidity sensor with those from the pneumotach for the (A) deep breathing area and (B) respiratory rate. The blue line indicates the bias, and the dotted lines indicate the limits of agreement.

Population-based Bland-Altman analysis was used for the participant data [66]. We used the mean difference and limits of agreement (LoAs) to quantify the humidity sensor-PNT correlation. Bland-Altman graph analysis is a simple way to assess the bias between the average differences and estimate an interval of agreement in which 95% of the sensor data differences fall relative to the PNT data. Both data sets were analyzed using unit difference and percent difference charts. Figure 7 shows the Bland-Altman analysis for the DBA-normalized plots to help estimate the within-subject and between-subject variability. Bias and variability terms were fixed to zero when they did not increase the objective function by more than 2 points, and bias and variance variability were assumed to be normal and log-normally distributed [66]. The DBA's overall analysis yielded a bias of 0.03 with LoAs of –0.25 and 0.33. The RR bias was 0.018, and the LoAs were –1.89 and 1.89.

### Statistical Analysis

**Figure 8:**
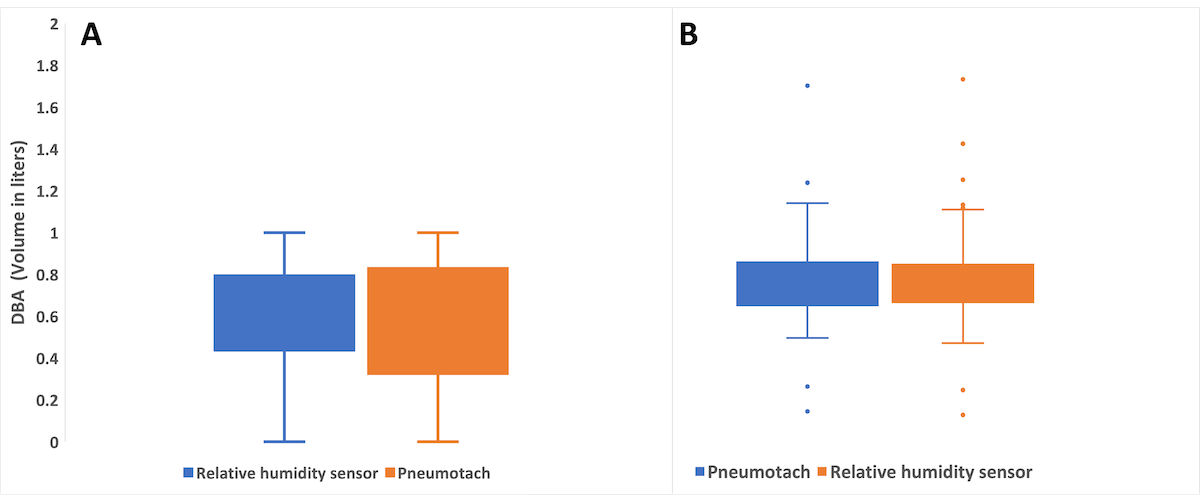
Box plots comparing the calculated (A) deep breathing area (DBA) and (B) respiratory rate values from the relative humidity sensor prototype with those from the pneumotach for all participants in the test data set.

We compared the PNT's experimental DBA and RR data with our RHS-based IoT prototype. Figure 8 shows box plots comparing the medians and IQRs from the commercial PNT with those from our prototype. The average DBA values from the PNT and our sensor were 0.56 (SD 0.29) and 0.59 (SD 0.28), respectively, showing similar values. The average RR with PNT was 17.61 (SD 1.73), and the average RR with our prototype was 17.58 (SD 1.71). This shows that our IoT sensor can accurately measure the participants' respiratory parameters.

Furthermore, the 1-way ANOVA analysis was useful to compare the similarities between the DBA and RR data sets, as shown in Table 1 [64]. After reviewing the ANOVA results, we still needed to understand subgroup differences among the different experimental and control groups. For the ANOVA, we used an optimal *P* value of .05 to test the hypothesis. The results showed values of *F*_1,39_=0.016 and *F*_1,347_=0.01, indicating that the differences between the group averages were negligible [67]. We could not reject the null hypothesis because the corresponding *P* values of .64 for DBA and .89 for RR were greater than .05 [67]. Thus, we concluded that there were no statistically significant differences between the mean DBA and RR measurements taken by the commercial PNT and our IoT sensor prototype.

**Figure 9:**
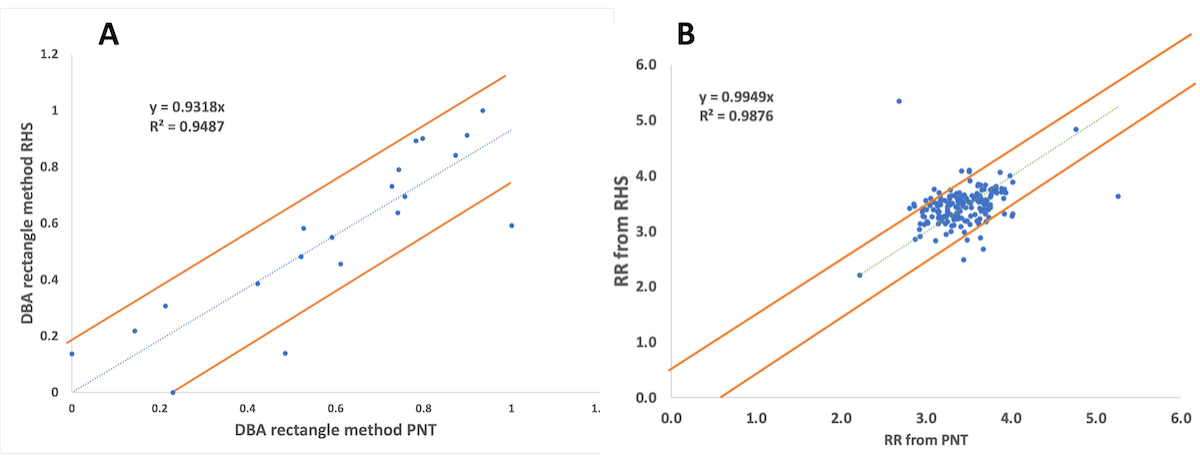
The plot shows the root mean square error (RMSE) of the (A) deep breathing area (DBA) and (B) respiratory rate (RR) values, comparing our relative humidity sensor (RHS) prototype against the commercial pneumotach (PNT).

**Figure 10:**
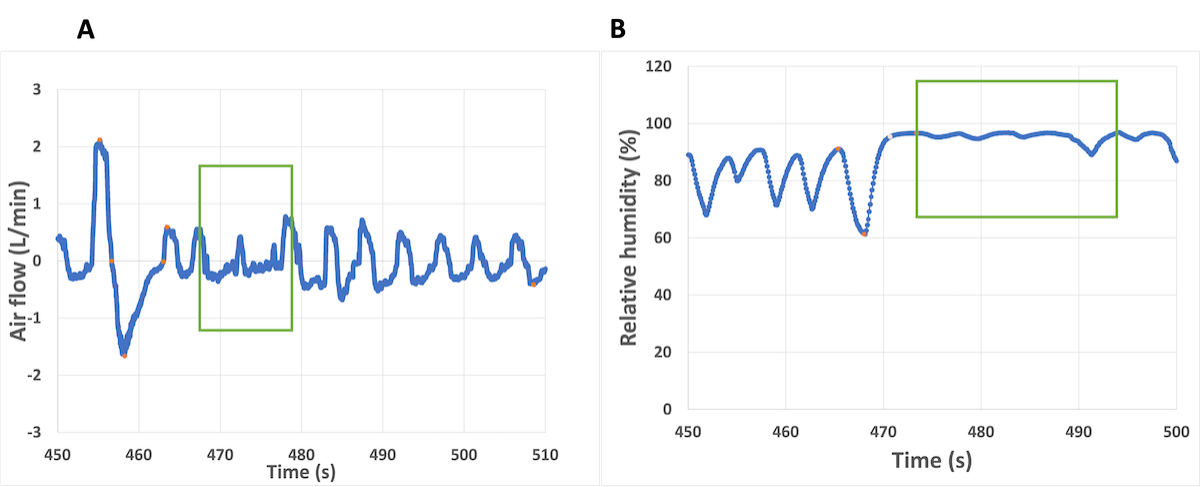
Two examples of breathing pattern anomalies during the deep breathing area and respiratory rate measurements using (A) the commercial pneumotach device and (B) our own relative humidity–based sensing device. The maximum point shows the value of the inspiratory reserve volume, while the minimum represents the expiratory reserve volume during deep breathing measurements; during a normal breathing pattern, the graph shows the tidal volume.

Finally, Figure 9 shows the root mean square error (RMSE) for the DBA and RR measurements, when comparing the commercial PNT with our RHS prototype. The trend includes the highest and lowest RR values. Abnormal breathing patterns during DBA and RR measurements cause data anomalies. The PNT and RHS prototype anomalies are shown in Figure 10. Errors in the DBA and RR can also be caused by variations in the participant's breathing pattern during the test [65]. This pattern appeared only once during our measurement, represented by the outlier points outside of the RMSE and DBA regions present in Figure 10. Most devices are very sensitive to changes in breathing patterns, especially during flow measurements because the face mask makes it hard to breathe normally. It is possible to ameliorate this error by averaging the volume and RR measurements over a longer period. Future generations could leverage sensor fusion, multitenancy (sequential usage of different sensors), or deep learning predictive value structures to continuously monitor patients' vital signs [68]. After examining the volumes (ERV, IRV, TV, VC) and RR measurements, we concluded that our IoT RHS-based device offers a unique way to properly measure essential respiratory parameters using a low-cost sensor and without heavy-duty medical devices.

**Table 1:** Results from the 1-way ANOVAs of respiratory volume and rate measurements.

Source of variation		SS^a^	*df*	MS^b^	*F*	*P* value
**Respiratory volume (L)**						
	Between groups	0.02	1	0.014	0.016	.68
	Within groups	3.13	38	0.08	—^c^	—
	Total	3.15	39	—	—	—
**Respiratory rate (beats per minute)**						
	Between groups	0.002	1	0.001	0.01	.89
	Within groups	39.09	346	0.11	—	—
	Total	39.09	347	—	—	—

^a^SS: sum of squares.

^b^MS: mean squares.

^c^Not applicable.

## Discussion

### Principal Findings

First, we showed that we can measure respiratory parameters using the proposed RHS-based device. To calculate the TV, we used the area under the RH curve using the rectangle method, as described. Second, we compared the measurements from the proposed device with those from the gold standard PNT for all participants. The Bland-Altman analysis showed that the measurements were within the LoAs and the bias was very low. Furthermore, the results from the ANOVA indicated that the *P* values were above the threshold value of *P*<.05*.* Both statistical experiments confirmed that the group average measurements from the PNT and our proposed device were similar within statistical limits (ie, we can use our proposed device to measure respiratory parameters with an accuracy that is close to that of the gold standard). This is further corroborated by the box plots of the DBA and RR values. Most importantly, our device is 100 times [61,62] less expensive than the PNT device.

### Comparison With Prior Work

The literature suggests that continuous and precise lung volume and RR monitoring is difficult. Lung volume, a critical indication of COPD status and development, cannot be assessed by RR alone [34]. TV, ERV, IRV, and VC can indicate respiratory depression. Researchers have measured lung volume with a spirometer, but it is inaccurate, has a large latency, and cannot be used while the patient is asleep [35]. Our method improves continuous lung volume monitoring by not requiring the patient to be supine. It also measures lung volume without a costly sensor or imaging tests.

### Strengths and Limitations

Our device is a low-cost medical device. It provides an opportunity to measure respiratory parameters in real time. The device uses a widely used and tested commercial humidity sensor that is stable and has a high signal-to-noise ratio. We tested the device with a variety of participants with different demographic characteristics and of different sexes to ensure that the results are reproducible. Despite all the strengths, there are certain limitations to our device. First, the framework of our device is not suitable for commercial applications as there is a large number of ad hoc components. There is scope to improve the design of the device by incorporating the sensors in the fabrication of the mask. Second, detailed study of the device with a larger number of participants and varying environmental conditions is required for further testing and calibration.

### Future Work

Due to the restrictions inherent to variations in breathing patterns and device sensitivity, we foresee the development of our own array of humidity sensors, coupled with deep learning data processing, with the goal of resolving any problems associated with breathing pattern deviations. This will enable extrapolation of the unusable measurements to ensure the same level of precision as linear results. This array of humidity sensors printed on a flexible base would permit the incorporation into materials for surgical masks without affecting the usability. Limitations may include a high-humidity environment, which may increase the risk of saturating the humidity sensor. A combination of pressure and temperature measurements would limit this bias. Ideally, the sensor should be integrated into the mask so that it can be used in real-life conditions, without obstructing the ventilatory connection. Increasing the sample size will not validate the use of the sensor, but the inclusion of profiles with respiratory disorders would make it possible to obtain measurements with less obvious variations in amplitude and thus include situations in which respiratory disorders could be detected [12].

### Conclusions

Wearable IoT medical technologies are developing as viable options not just to monitor patients at home after hospitalization but also to boost the affordability and accessibility of quality health care. In fact, the development of more effective and less expensive wearable medical devices could allow patients to monitor their health at home. In recent years, the number of wearable medical devices for chronic disease monitoring has expanded. Last, improved medically certified sensors will facilitate the development of better and less expensive medical IoT devices. In our investigation, we utilized a single sensor to simultaneously detect pressure, temperature, and humidity. The literature indicates that temperature is frequently used to measure RR [45]. The results indicate that our RHS can measure RR within statistically acceptable control limits. The results of the 1-way ANOVA indicate that the group means of the PNT are equivalent to our RHS within the standard margin of error of the instrument. This is further supported by the Bland-Altman analysis, which revealed low values of 0.03 and 0.018 for the bias and RR, respectively. The data analysis revealed that the evolution of RRs over time for the PNT and our low-cost RHS follows a similar pattern. This is, as far as we are aware, the first study to investigate the use of RHS for reliably monitoring respiratory volumes on a medical IoT platform. We plan to examine the applicability of the RHS sensor to detect more complex respiratory disorders using deep learning in a future phase of development.

In terms of utility, it is evident that continuous and precise monitoring of lung capacity and RR represents a significant basic obstacle. Nonetheless, it presents a tremendous opportunity to monitor patients with OSA in intensive care or during surgery. Lung volumes, namely TV, can be utilized to manage respiratory pauses or identify the breathing pattern in patients with OSA who utilize C-PAP or A-PAP devices, which, when combined with artificial intelligence, can detect apnea occurrences and enhance A-PAP performance. Regarding ventilated patients, assessing TV will allow for better control of ventilatory weaning.

## Abbreviations

A-PAP: automatic positive airway pressure
COPD: chronic obstructive pulmonary disease
C-PAP: continuous positive airway pressure
DBA: deep breathing area
ECG: electrocardiography
ERV: expiratory reserve volume
ICU: intensive care unit
IoT: Internet of Things
IRV: inspiratory reserve volume
LoA: limit of agreement
OSA: obstructive sleep apnea
PNT: pneumotach
PPG: photoplethysmography
RH: relative humidity
RHS: relative humidity sensor
RMSE: root mean square error
RR: respiratory rate
TV: tidal volume
VC: vital capacity

## Data Availability

Data will be provided upon reasonable request.
